# A Complex Story of Multi-Species Vector–Malaria Parasite Interactions and Seasonality in Burkina Faso

**DOI:** 10.4269/ajtmh.26-0442

**Published:** 2026-08-06

**Authors:** Colin J. Sutherland

**Affiliations:** ^1^ Department for Infection Biology, London School of Hygiene & Tropical Medicine, London, United Kingdom

The West African Sahel consistently sustains transmission of five human-infecting *Plasmodium* species, including *Plasmodium falciparum*, the dominant species in Africa, and four others^1^, delivered to the bloodstream of their prospective mammalian hosts by three major anthropophilic mosquito species of the *Anopheles gambiae* complex, plus *A. funestus*.^2^ The region is characterized by a long dry season in which mosquito numbers plummet and malaria transmission levels fall dramatically. The timing and duration of the rain-dependent transmission season, which vary among different settings, can fluctuate substantially from one year to the next in a particular location.^3^ These three sources of variation and diversity – parasite species, vector species, and climate – together generate multi-factorial complexity in Sahelian malaria transmission. This fascinating picture conjures up a series of important questions that are difficult to address. How is the relative abundance of different vectors affected by the timing, duration, and intensity of each rainy season? Are some vectors predominant only at certain stages of the season? Do the various plasmodial species display the same relative prevalences in each of the vector species as they do among human hosts?

In this issue of *The American Journal of Tropical Medicine and Hygiene,* Lado et al.^4^ present an intriguing analysis of *Plasmodium* species carriage by human and mosquito hosts in a high malaria burden setting in southwestern Burkina Faso. Their unique sampling strategy, conducted under protocols designed for randomized studies of ivermectin impact, has generated an informative set of secondary outputs, which are observational in nature, but start to address the questions raised above. Study samples were from the RINDAMAL trial, conducted during the July to November wet season of 2015, and the RINDAMAL II trial, conducted from July to November in 2019 and 2020, with both studies in Diegbougou District.

Among 66 individuals who participated in the 2015 study and presented with fever, some of whom were sampled on multiple occasions, *P. falciparum* monoinfections were confirmed in 42% of the 175 blood samples tested. Also, seven *P. vivax* infections and a single *P. ovale* spp. infection were identified. In the two seasons of the RINDAMAL II trial, a different picture was seen. Of 405 peripheral blood samples tested from 70 randomly selected participants with fever, 225 (56%) were positive for *Plasmodium* spp. DNA, including 208 (92%) *P. falciparum*, alone or mixed, 46 (11%) *P. malariae* infections (alone or mixed), 23 (6%) *P. ovale* spp. infections (alone or mixed) and a single *P. vivax* infection, which was mixed with *P. falciparum*. Thus, although *P. falciparum* prevalence was similar between the two study periods, both *P. malariae* and *P. ovale* spp. parasitemia were significantly more common in the 2019–2020 study.

Entomological data also offered some surprises. First, 99% of the 2,801 *Anopheles* spp. mosquitoes captured indoors in 2015 were identified as *A. gambiae* s.l., whereas in the 2019–2020 study, among 7,942 indoor caught mosquitoes, this proportion had fallen to 85%, the remaining 15% being *A. funestus*, almost all of which were caught in 2019. Thus, the relative abundance of *A. funestus* fluctuated among seasons. Second, the qPCR-confirmed *Plasmodium* sporozoite rate from control and ivermectin clusters in 2019–2020 was much higher in *A. funestus* (20.4%, *n* = 113, and 11%, *n* = 136, respectively) than *A. gambiae* (5.3%, *n* = 1,399, and 4.3%, *n* = 1,129). Remarkably, *A. funestus* predominantly harbored *P. ovale* spp., accounting for 63.2% of sporozoite-positive mosquitoes, compared to a much lower relative abundance in *A. gambiae*, in which *P. falciparum* accounted for the majority of infected salivary glands. *P. malariae* sporozoites were present in both vectors, accounting for 8% to 18% of positive salivary glands.

Taken together, these data present a difficult puzzle for those planning malaria control interventions in the West African Sahel. Each season differs not only in intensity of malaria transmission, but also as to whether or not *A. funestus* will be a significant contributor. If abundant, this latter vector species appears, based on these new data, to favor transmission of *P. ovale* spp., with unknown impact on overall morbidity, but in other seasons when *A. funestus* is scarce there may be little transmission of *P. ovale* spp. To add to this intricate interplay between parasite, vector, and human host, estimates of species-specific parasite abundance based on blood sampling do not match observations in mosquitoes. Blood samples indicate a higher prevalence of *P. malariae* compared to *P. ovale* infections, whereas entomological data indicate that *P. ovale* is the far more frequent parasite in the salivary glands of human-feeding mosquitoes. This is biologically interesting, of course, and is consistent with the evidence that travelers returning to non-endemic settings are more likely to present with *P. ovale* than *P. malariae* infection. This finding is presumably because *P. malariae* is less likely to transmit despite its greater abundance in human infections.^5^ Essentially, *P. ovale* is sustained through efficient mosquito transmission and a reservoir of dormant liver-stage hypnozoites, while *P. malariae* maintains long-term persistence at low densities in humans, with less frequent mosquito transmission.

The work of Lado et al.^4^ illustrates very well the value of long-term studies in a defined locality, deploying imaginative longitudinal sampling of multiple parasite stages in both humans and mosquitoes. The authors have presented us with a tantalizing descriptive analysis of this system, and its plasticity under the influence of varying rainfall patterns and likely other factors that impact year-to-year trends. With the current era of climate instability and global warming presenting the threat of even greater instability in rainfall patterns, these approaches to careful measurement of malaria entomological indices provide an invaluable blueprint for planning future studies to inform optimal malaria control practices.
